# Toward Evidence-Informed Staging in Septic Revision Total Knee Arthroplasty: One-Stage, 1.5-Stage, or Two-Stage?

**DOI:** 10.7759/cureus.114885

**Published:** 2026-08-20

**Authors:** Mathias S Salazar, Lisbeth T Almeida, Carlos A Moyano, Gustavo P Cadena

**Affiliations:** 1 Orthopedics and Traumatology, Novaclínica Santa Cecilia, Quito, ECU; 2 Orthopedics and Traumatology, Pontificia Universidad Católica del Ecuador, Quito, ECU; 3 Orthopedics, Hospital General Docente de Calderón, Quito, ECU; 4 Orthopedic Surgery, Novaclínica Santa Cecilia, Quito, ECU; 5 Trauma and Orthopedics, Veris Centrales Médicas, Quito, ECU

**Keywords:** 1.5-stage revision, articulating spacer, one-stage revision, periprosthetic joint infection, septic revision, total knee arthroplasty, treatment algorithm, two-stage revision

## Abstract

Periprosthetic joint infection (PJI) after total knee arthroplasty (TKA) remains one of the most consequential complications in adult reconstruction because successful treatment requires simultaneous infection control, restoration of a mechanically durable knee, preservation of function, and minimization of surgical morbidity. Two-stage exchange has traditionally been regarded as the reference strategy for chronic knee PJI, but contemporary comparative evidence increasingly challenges its routine use as the default for all patients. This narrative review synthesizes evidence from systematic reviews, meta-analyses, comparative cohorts, propensity-matched analyses, and decision-analytic studies evaluating one-stage, 1.5-stage, and two-stage septic revision TKA. The available data indicate that infection control after one-stage revision is broadly comparable with, and in some analyses superior to, two-stage revision in carefully selected patients. Conversely, two-stage exchange retains a central role in patients with severe host compromise, complex bone or soft-tissue defects, multidrug-resistant or fungal infection, uncertain microbiology, and reconstructive instability. The 1.5-stage strategy has emerged as a pragmatic function-preserving alternative, with encouraging infection-free survivorship and lower treatment burden; however, its long-term mechanical durability remains incompletely defined, particularly because aseptic loosening is consistently identified as a key risk. Evidence-based staging should therefore move beyond a binary hierarchy and toward an algorithm integrating host status, organism profile, soft-tissue envelope, bone loss, fixation requirements, prior failure, timing, and patient goals. Current evidence supports individualized multidisciplinary decision-making rather than routine use of any single staging strategy, while highlighting the need for prospective knee-specific studies using standardized definitions, patient-reported outcomes, mechanical survivorship, and cost-effectiveness endpoints.

## Introduction and background

Periprosthetic joint infection (PJI) after total knee arthroplasty (TKA) remains one of the most morbid and resource-intensive complications in adult reconstruction. Unlike aseptic revision, septic revision must simultaneously address biofilm-associated infection and restore a stable, functional, load-bearing knee. Two-stage exchange has historically been considered the standard strategy for chronic PJI, involving implant removal, extensive debridement, placement of an antibiotic-loaded spacer, systemic antimicrobial therapy, and subsequent reimplantation. Although indispensable in many complex infections, this approach exposes patients to prolonged disability, spacer-related complications, repeated surgery, uncertain progression to reimplantation, and substantial treatment burden.

Contemporary evidence has challenged the assumption that a greater number of operative stages necessarily provides superior infection control. Knee-specific and broader arthroplasty meta-analyses have reported similar or, in some analyses, lower reinfection rates after one-stage revision compared with two-stage exchange, without consistent differences in reoperation or mortality [1-4]. However, these findings remain difficult to interpret because the underlying literature is heterogeneous and predominantly observational. Conversely, other knee-specific analyses have reported greater infection eradication success with two-stage revision, further demonstrating that conclusions depend on study selection, outcome definitions, and patient characteristics [5,6]. Moreover, the limitations of two-stage treatment extend beyond reinfection: a large systematic review of planned two-stage exchange reported infection eradication of approximately 74%, reinfection of 15.7%, and failure to proceed to reimplantation in 16.9% of patients [5].

A major source of uncertainty is selection bias. Patients undergoing one-stage revision are frequently selected because of favorable host physiology, identifiable and antibiotic-susceptible organisms, adequate soft-tissue coverage, and reconstructable bone defects, whereas two-stage exchange is more commonly used in patients with resistant organisms, sinus tracts, severe bone loss, immunocompromise, or previous treatment failure. Comparative studies therefore suggest that outcome is determined not only by staging strategy but also by host, microbiological, and local reconstructive factors. Matar et al. reported comparable infection control between one- and two-stage revision while identifying advanced age and sinus tract as predictors of failure independent of staging strategy [7]. Proposed criteria for one-stage exchange have similarly emphasized host status, organism susceptibility, and the ability to achieve adequate soft-tissue and osseous reconstruction [8], while other studies have highlighted interactions between microbiological difficulty and mechanical reconstructive demands [9]. Even culture-negative PJI, traditionally considered unfavorable for single-stage treatment, may not uniformly preclude its use in appropriately selected patients [10].

Patient-centered outcomes further complicate comparisons based exclusively on infection eradication. Propensity-matched evidence has demonstrated favorable patient-reported outcomes after one-stage revision without an associated increase in reinfection or readmission [11], and reviews have generally reported functional outcomes that are comparable or occasionally superior to those of two-stage exchange [12-14]. Nevertheless, heterogeneity in outcome measures, follow-up, and reporting prevents definitive conclusions regarding functional superiority.

The emergence of 1.5-stage revision has further expanded the treatment spectrum. This strategy uses an articulating prosthetic spacer intended for prolonged or potentially indefinite retention, with subsequent revision reserved for recurrent infection, pain, mechanical failure, or functional deterioration. Available pooled evidence suggests favorable infection control compared with conventional two-stage exchange but also identifies concerns regarding aseptic loosening [15]. Systematic reviews have reported infection-free success of approximately 86.8% and potential advantages related to pain, complications, and cost. However, current evidence is limited by heterogeneous techniques and predominantly short- to mid-term follow-up [16,17]. Accordingly, 1.5-stage revision should be considered a distinct reconstructive strategy rather than simply an incomplete two-stage pathway.

Despite an expanding literature on all three strategies, an important knowledge gap remains: there is no well-validated, patient-stratified framework that reliably integrates host physiology, organism characteristics, local soft-tissue and bone conditions, reconstructive complexity, and patient-centered treatment priorities to determine when one-stage, 1.5-stage, or two-stage revision is most appropriate. Direct comparisons are limited by selection bias, heterogeneous definitions of treatment success, variable surgical techniques, and differences in case complexity. This narrative review therefore synthesizes the available evidence to develop an evidence-informed framework for staging septic revision TKA, with the aim of identifying the clinical circumstances in which each strategy may offer the most appropriate balance between infection control, reconstructive durability, functional recovery, and treatment burden.

This narrative review synthesized 50 studies identified through PubMed/MEDLINE and Embase, prioritizing clinically relevant evidence on one-stage, 1.5-stage, and two-stage revision strategies for chronic PJI after TKA.

## Review

Methodology

This study was conducted as a narrative review. Relevant literature was identified through searches of PubMed/MEDLINE and Embase, and 50 articles were included in the final evidence synthesis. Studies were selected according to their relevance to septic revision TKA and to one or more of the following five prespecified domains: comparative outcomes among one-stage, 1.5-stage, and two-stage revision; patient selection according to host, pathogen, and local factors; indications and outcomes for 1.5-stage revision; timing and technical strategy during staged exchange; and decision-making frameworks.

Priority was given to knee-specific systematic reviews and meta-analyses, comparative cohort studies, propensity-matched analyses, decision-analytic models, and studies explicitly evaluating chronic PJI after TKA. Hip and knee studies were included when they provided relevant knee subgroup data or when they addressed 1.5-stage revision, for which knee-specific comparative evidence remains limited. Studies were selected based on clinical relevance, applicability to the predefined review domains, and their contribution to the interpretation of staging strategies rather than according to a formal systematic review protocol.

The synthesis was narrative rather than a de novo systematic review because the objective was to integrate the available literature and provide a clinically oriented interpretation of current staging strategies. Outcomes of interest included reinfection, infection eradication or infection-free survivorship, reoperation, complications, mortality, reimplantation failure, function, patient-reported outcomes, pain, spacer retention, aseptic loosening, timing of reimplantation, and cost-effectiveness.

Evidence was interpreted with explicit attention to study design, joint specificity, patient selection criteria, heterogeneity in definitions of success and failure, follow-up duration, and potential allocation bias. Contradictory findings were not resolved by simple vote counting; instead, they were interpreted in relation to differences in patient selection, surgical technique, microbiology, spacer construct, timing, and outcome definitions. The resulting framework should therefore be understood as an evidence-informed staging approach rather than a formal clinical guideline. It is intended to support multidisciplinary assessment by arthroplasty, infectious disease, plastic surgery, microbiology, anesthesia, and rehabilitation teams and should not replace individualized clinical judgment.

Results

Comparative Infection Control and Patient-Centered Outcomes

Across contemporary comparative evidence, one-stage revision appears at least comparable with two-stage exchange for infection control in selected patients with chronic septic TKA, although the direction and magnitude of effect vary by study design. The knee-focused meta-analysis by Xie et al. found a statistically significant reduction in reinfection after one-stage revision, whereas the earlier meta-analysis by Kunutsor et al. found nearly equivalent reinfection rates [1,2]. Goud et al. provided a broader benchmark by showing knee reinfection of 12.7% after one-stage and 16.2% after two-stage revision, with the important limitation that one-stage knee studies were fewer and more selected [3]. Sun et al. extended this pattern by reporting lower reinfection and complication rates after one-stage revision without significant differences in reoperation or mortality [4,5]. Taken together, these findings weaken the assumption that two-stage exchange is intrinsically superior in all chronic knee PJI cases. The evidence remains inconsistent. Patel et al. reported higher infection eradication success after two-stage revision in a knee-specific meta-analysis, with reinfection rates of 24.1% after one-stage and 14.0% after two-stage revision [6]. This discrepancy likely reflects narrower inclusion criteria, variation in definitions of success, and heterogeneity in surgical indications. More importantly, most comparative studies are retrospective, and two-stage cohorts often contain biologically and mechanically more complex cases. Consequently, pooled comparisons should not be interpreted as direct proof that one strategy is universally superior. Two-stage exchange remains an important comparator, but it should not be treated as a risk-free standard. Piuzzi et al. showed that planned two-stage exchange carries a substantial burden: approximately one in six patients did not undergo reimplantation, and knee patients had worse Musculoskeletal Infection Society outcomes and higher mortality than hip patients [5]. This finding reframes two-stage exchange as a high-burden pathway rather than simply a safer default. Functional data also support a more nuanced interpretation. Klemt et al. found better patient-reported outcomes after single-stage revision TKA with similar reinfection and readmission, while other reviews report broadly comparable or better function after one-stage revision despite inconsistent patient-reported outcome measure reporting [11-14]. Infection eradication should therefore not be the only endpoint used to select a staging strategy; treatment completion, morbidity, function, quality of life, and patient preference are clinically decisive.

Patient Selection: Host, Pathogen, and Local Reconstructive Factors

The most consistent finding across studies is that patient selection modifies the apparent effectiveness of each staging strategy. Classical criteria favoring one-stage revision include a host without severe immunocompromise or systemic sepsis, a known organism susceptible to available systemic and local antibiotics, adequate soft tissue permitting primary closure, and bone stock sufficient for stable reconstruction [8]. These criteria are supported by comparative cohort evidence showing that failure is driven by variables such as sinus tract, advanced age, and revision complexity rather than by staging alone [7,8]. In this context, one-stage revision is best viewed as an aggressive but rational option when radical debridement, immediate reconstruction, and targeted antimicrobial therapy can be performed with high confidence. Pathogen-related variables remain central but should not be applied simplistically. Difficult-to-treat organisms, multidrug resistance, fungal infection, and polymicrobial infection frequently shift decision-making toward two-stage exchange because they increase uncertainty regarding antimicrobial effectiveness and adequacy of source control [8,9,13]. However, culture-negative infection alone should not automatically exclude one-stage revision. Van den Kieboom et al. found similar outcomes between one-stage and two-stage revision for chronic culture-negative PJI in a selected cohort, suggesting that diagnostic sampling quality, clinical phenotype, local tissue status, and antimicrobial planning may matter more than culture status in isolation [10]. Local reconstructive factors often determine feasibility. Severe bone loss, compromised extensor mechanism, poor soft-tissue envelope, complex sinus tracts, anticipated need for flap coverage, and inability to achieve stable fixation generally favor two-stage exchange, or another staged reconstructive plan [9,13]. Anderson Orthopaedic Research Institute type III defects, weak soft tissues, and high revision complexity cases are repeatedly described as reasons to avoid routine one-stage revision. Conversely, adequate soft tissue and limited-to-moderate bone loss support one-stage or 1.5-stage strategies. The evidence therefore supports a host-pathogen-local triad: one-stage revision is strongest when all three domains are favorable; two-stage exchange is preferred when two or more domains are unfavorable; and 1.5-stage revision occupies an intermediate space when infection control and function may be achieved with a durable articulating construct but automatic reimplantation is not desirable.

The 1.5-Stage Strategy: Promise, Trade-Offs, and Uncertainty

The 1.5-stage concept has emerged because some patients treated with articulating spacers either do not proceed to or may not require formal reimplantation. Siddiqi et al. described the technique as a prosthetic articulating spacer intended for definitive or prolonged use, with second-stage reconstruction reserved for recurrent infection, mechanical failure, or unacceptable function [18]. Hernandez et al. reported that most patients retained the spacer at short- to mid-term follow-up, with recurrent PJI in approximately 10% [19]. Nabet et al. found infection-free survival of 85.1% after 1.5-stage revision versus 75.0% after two-stage revision in a comparative TKA series, whereas Belay et al. reported similar infection clearance between permanent-spacer and two-stage cohorts after propensity matching [20,21]. These findings indicate that prolonged spacer retention may function as a definitive treatment strategy in selected patients rather than exclusively as a delayed pathway toward second-stage reimplantation.

Pooled evidence is encouraging but remains limited. Ibrahim et al. reported lower reinfection after 1.5-stage compared with two-stage revision, with no significant differences in overall eradication, complications, readmission, dislocation, or periprosthetic fracture [15]. Khnanisho et al. reported a mean infection-free success rate of 86.8% and infection-related failure of 12.6%, with several comparative studies demonstrating similar or better infection-free survivorship than two-stage exchange [16]. Festa et al. reported infection control of 84.6% after 1.5-stage revision compared with 76.1% after two-stage revision, with lower recurrence in the 1.5-stage group [17]. However, these findings should be interpreted cautiously. The available literature is characterized by relatively small cohorts, predominantly retrospective study designs, limited follow-up, potential dependence on institutional expertise, and heterogeneity in spacer and implant constructs. These methodological and technical differences limit the generalizability of reported outcomes and preclude definitive conclusions regarding the comparative effectiveness of 1.5-stage revision.

Mechanical durability represents an additional source of uncertainty. Ibrahim et al. identified a substantially higher risk of aseptic loosening after 1.5-stage revision [15]. Festa et al. similarly identified conversion to a definitive prosthesis and aseptic loosening among the leading causes of reoperation [17]. Although short-term radiographic series have demonstrated acceptable construct stability, the absence of long-term follow-up limits confidence regarding durable implant survivorship [19,20]. Consequently, favorable short- and mid-term infection control results should not be assumed to predict long-term mechanical success.

The 1.5-stage strategy should therefore not be regarded as a low-risk shortcut or as a universally applicable alternative to conventional staged exchange. Its potential advantages include avoidance of an obligatory second operation and preservation of function during infection treatment, but these benefits must be weighed against uncertainty regarding long-term fixation and implant survivorship. Current evidence supports consideration of 1.5-stage revision in carefully selected patients, particularly when additional surgery carries substantial burden and when local bone and soft-tissue conditions permit a stable articulating construct. However, broader adoption should remain cautious until larger comparative cohorts, more standardized reconstructive techniques, and longer-term follow-up provide greater confidence regarding durability and patient selection.

Timing, Spacers, Antibiotics, and Algorithmic Decision-Making

For patients treated with classic two-stage exchange, timing is not a neutral variable. Puetzler et al. found that time to reimplantation exceeding 94 days after static spacer placement was associated with significantly increased reinfection risk [22]. Vielgut et al. similarly reported worse infection outcomes when spacer retention exceeded approximately 83 days and suggested that reimplantation within 12 weeks, once infection control is achieved, may be advantageous [23]. These findings do not imply that every patient should undergo early reimplantation regardless of clinical status, but they challenge indefinite spacer retention as the default interstage strategy when the plan is formal two-stage exchange. Prolonged intervals may reflect host frailty, persistent infection, poor soft tissue, or logistic delays, but they may also contribute to morbidity and recurrent infection risk. Spacer design and local antibiotic delivery further influence outcomes. Articulating spacers are generally associated with better preservation of motion and may improve function compared with static spacers, while static spacers remain useful for severe instability, extensor mechanism compromise, massive bone loss, or poor soft tissues. Vasarhelyi et al. and Romano et al. support the importance of spacer selection, with articulating spacers often providing functional advantages and, in some analyses, improved infection eradication [24,25]. Antibiotic selection should be organism-directed whenever possible, compatible with cement elution properties when local therapy is used, and coordinated with systemic therapy by infectious disease specialists. The algorithmic implication is that two-stage revision is not one operation but a sequence of decisions: debridement quality, spacer type, antibiotic strategy, monitoring criteria, timing of reimplantation, and reconstructive plan all contribute to success. Decision-analytic work supports moving from tradition to structured algorithms. Srivastava et al. found one-stage revision economically favorable in chronic knee PJI patients without compelling indications for two-stage exchange, highlighting that cost-effectiveness depends strongly on infection control, reoperation, and treatment completion [26]. More recent studies evaluating one-stage eligibility and two-stage outcomes suggest that classical one-stage indications may predict better survivorship even when patients undergo two-stage exchange, indicating that eligibility criteria may reflect baseline prognosis rather than procedure-specific benefit [27,28]. Multistage salvage procedures can achieve remission in difficult cases but with high revision burden and mortality, emphasizing that repeated staging should be reserved for selected salvage indications rather than considered a routine extension of failed two-stage exchange [29-31]. The evidence therefore supports an algorithm that first identifies contraindications to immediate reconstruction, then determines whether functional preservation with a 1.5-stage construct is feasible, and finally reserves formal two-stage exchange for cases in which infection biology or reconstructive uncertainty makes immediate definitive reconstruction unsafe.

Discussion

The central finding of this review is that staging in septic revision TKA is better conceptualized as a multidimensional decision rather than as a hierarchy in which one strategy is inherently superior. One-stage, 1.5-stage, and two-stage revision should instead be considered alternative approaches whose suitability depends on host status, microbiology, local reconstructive conditions, mechanical requirements, and patient goals. Although several comparative studies have reported outcomes after one-stage revision that are comparable with or numerically more favorable than those after two-stage exchange, these observations arise predominantly from retrospective, nonrandomized populations and are highly susceptible to selection bias and residual confounding [1-4,7,11]. Patients selected for one-stage revision often have more favorable host, microbiological, and local conditions, whereas more complex infections are preferentially treated with staged exchange. Conversely, studies including less selectively chosen one-stage populations or using different definitions of success have reported more favorable results for two-stage revision [6]. Current evidence therefore cannot establish the superiority of one staging strategy over another; rather, it supports shifting the clinical question toward which combination of risk factors makes immediate definitive reconstruction reasonable, uncertain, or inappropriate. Host assessment is a fundamental component of this decision. Systemic sepsis, uncontrolled immunosuppression, severe malnutrition, uncompensated diabetes, major vascular insufficiency, and limited physiological reserve may compromise infection control, wound healing, or tolerance of reconstruction. Classical one-stage selection criteria emphasize a relatively favorable host, and contemporary evidence similarly suggests that host characteristics influence outcomes across treatment strategies [8,27,32-34]. However, comorbidity alone does not establish that two-stage exchange is preferable. Frail patients may also be poorly suited to the cumulative burden of two major procedures, making prolonged spacer retention or a 1.5-stage strategy potentially relevant when adequate debridement and stable articulation are achievable. Host assessment should therefore address both the biological feasibility of infection control and the patient’s ability to tolerate the proposed treatment pathway.

Microbiology represents a second major risk domain. Identification of an antibiotic-susceptible organism may facilitate targeted systemic and local antimicrobial therapy and can support consideration of one-stage revision, whereas resistant, fungal, polymicrobial, and other difficult-to-treat organisms introduce greater uncertainty and are frequently managed with staged exchange, particularly when adverse host or local factors coexist [8,9,13,32,33]. These associations should not be interpreted as absolute indications. Culture-negative infection, for example, is not necessarily equivalent to uncontrolled microbiology when diagnostic sampling has been adequate and other clinical conditions are favorable [10]. Likewise, identification of a susceptible organism does not compensate for an unfavorable host or a compromised reconstructive environment. Microbiology is therefore best regarded as one interacting determinant rather than a stand-alone staging rule.

Local reconstructive conditions are equally important. Immediate definitive reconstruction requires adequate implant and cement removal, thorough debridement, management of dead space, reliable soft-tissue closure, and the ability to achieve stable fixation. Severe metaphyseal bone loss, ligament insufficiency, extensor mechanism compromise, the need for complex soft-tissue coverage, or inability to obtain stable fixation may reduce the feasibility of one-stage exchange. In such circumstances, staged treatment can provide additional time for infection management and reconstructive planning. Conversely, when a stable articulating construct can be achieved but obligatory reimplantation would impose substantial additional burden, prolonged spacer retention or a 1.5-stage approach may be considered.

The emergence of 1.5-stage revision has broadened the available treatment spectrum by formalizing prolonged retention of a functional articulating construct. Reported series suggest that this strategy may reduce the need for an obligatory second procedure and preserve function in selected patients [15-21]. However, these findings should remain cautiously interpreted because the literature consists largely of relatively small retrospective cohorts, with short- to mid-term follow-up, heterogeneous implant constructs, and potential influence from institutional expertise. The principal unresolved concern is mechanical durability. Aseptic loosening and later conversion remain clinically important failure modes, and favorable early infection-control results cannot be assumed to predict long-term implant survivorship. Accordingly, 1.5-stage revision should be viewed as a selective strategy rather than as evidence-supported justification for broad replacement of conventional staged treatment.

Two-stage exchange remains an important option, particularly when infection biology is unfavorable, local tissues are compromised, bone loss requires staged reconstruction, difficult-to-treat organisms are present, previous revisions have failed, or diagnostic and antimicrobial uncertainty remains substantial. Its outcomes, however, should not be judged solely among patients who successfully complete both operations. Evidence regarding non-reimplantation and interstage complications indicates that death, persistent spacer retention, resection arthroplasty, arthrodesis, amputation, and failure to proceed to reimplantation are clinically relevant components of the treatment pathway [5]. This distinction is especially important in elderly or medically fragile patients, in whom the interstage period may itself represent a substantial source of morbidity.

Timing of reimplantation should likewise be interpreted cautiously. Associations between longer interstage intervals and poorer outcomes beyond approximately 83 to 94 days do not establish a universal temporal threshold because prolonged intervals may reflect more difficult infections, compromised hosts, or unresolved reconstructive problems [22,23]. Nevertheless, the available evidence does not support the assumption that longer delay is intrinsically protective. Once infection control, soft-tissue readiness, and medical optimization have been achieved, continued delay may expose patients to additional spacer-related and functional morbidity. Conversely, reimplantation before adequate infection and tissue control remains inappropriate. The clinically relevant principle is therefore readiness rather than adherence to a fixed interval.

Antimicrobial treatment should be integrated into staging decisions rather than considered separately from surgical strategy. PJI management principles emphasize organism identification, surgical source control, and coordinated antimicrobial therapy [31]. Immediate reimplantation requires sufficient confidence in microbiological diagnosis and antimicrobial planning, whereas staged and 1.5-stage strategies incorporate different expectations regarding local antibiotic delivery, duration of implant retention, and subsequent reconstruction. These decisions therefore require multidisciplinary coordination between arthroplasty surgeons, infectious disease specialists, and microbiology teams. Taken together, the evidence supports a flexible decision framework based on the following four interacting domains: confirmation of chronic PJI and feasibility of adequate debridement; host status; local reconstructive conditions; and microbiological risk. One-stage revision may be considered when these domains are favorable, but the available comparative evidence does not demonstrate that it is superior to two-stage exchange. Similarly, 1.5-stage revision may be considered when debridement and stable articulation are feasible but planned reimplantation is undesirable or uncertain, whereas two-stage exchange remains appropriate when microbiology, soft tissues, bone loss, host optimization, or previous treatment failure favor a staged pathway. These categories are evidence-informed rather than validated thresholds and should not be interpreted as a prescriptive algorithm.

The principal limitation of the current literature is the predominance of retrospective, nonrandomized studies. Differences in host characteristics, PJI definitions, organism profiles, spacer constructs, antibiotic protocols, interstage timing, follow-up, and definitions of treatment success create substantial clinical heterogeneity and residual confounding. Apparent differences in reinfection or functional outcomes between staging strategies may therefore reflect allocation and patient selection effects as much as treatment efficacy. Infection eradication itself is variably defined as absence of recurrent infection, absence of reoperation, implant retention, or composite outcome criteria, further limiting direct comparison. Functional outcomes and patient-reported measures are also inconsistently reported, while economic analyses remain highly dependent on healthcare system assumptions. Future research should prioritize prospective, knee-specific comparative studies with predefined stratification according to host, microbiological, and local reconstructive risk. Although randomized trials may be difficult because treatment allocation is strongly influenced by clinical characteristics and patient preferences, prospective multicenter cohorts or pragmatic registry-based designs could reduce some of the limitations of existing retrospective comparisons. Relevant outcomes should extend beyond reinfection to include reoperation, completion of reimplantation, mortality, patient-reported outcomes, range of motion, extensor mechanism function, spacer-related complications, aseptic loosening, antimicrobial adverse events, quality-adjusted life-years, and total treatment cost. For 1.5-stage revision, in particular, long-term mechanical survivorship at five to ten years remains a critical evidence gap.

Clinical Implications

Septic revision TKA should be staged according to risk domains rather than tradition alone. One-stage revision should be considered for patients with favorable host status, adequate soft tissue, manageable bone loss, stable reconstructive options, and known susceptible organisms. Two-stage exchange should remain preferred for difficult-to-treat pathogens, severe local compromise, uncertain source control, need for staged soft-tissue or bone reconstruction, and complex salvage cases. 1.5-stage revision should be considered when an articulating construct can provide acceptable function and stability, particularly in patients for whom avoiding an obligatory second operation is medically valuable or aligned with informed patient goals.

Limitations

This review is limited by the quality, design, and heterogeneity of the underlying evidence. Most included studies were retrospective and observational, and treatment allocation was rarely randomized, creating substantial susceptibility to selection bias, confounding by indication, and residual confounding. The absence of adequately powered randomized trials limits the ability to establish the comparative superiority of one-stage, 1.5-stage, or two-stage revision strategies. Publication bias may also influence the available literature, particularly if favorable institutional experiences or successful treatment strategies are more likely to be reported than negative or inconclusive outcomes.

Considerable heterogeneity further limits direct comparison among studies. Definitions of chronic PJI, infection eradication, reinfection, treatment failure, and reoperation vary substantially across the literature, and meta-analyses frequently combine studies using different diagnostic criteria and outcome definitions. Surgical techniques, debridement protocols, spacer constructs, fixation methods, antimicrobial regimens, and thresholds for reimplantation are also heterogeneous. In addition, outcomes may be influenced by differences in institutional volume, multidisciplinary resources, and surgical expertise, which may limit the generalizability of results obtained at specialized centers. Evidence for 1.5-stage revision remains particularly immature, with relatively small cohorts, predominantly retrospective designs, frequent inclusion of mixed hip and knee populations, heterogeneous implant constructs, and mainly short- to mid-term follow-up. Consequently, long-term mechanical durability and implant survivorship remain uncertain.

Finally, because this narrative review synthesized a curated evidence set rather than performing a de novo systematic review with formal study selection, risk-of-bias assessment, or quantitative evidence grading, it may not include every relevant study and cannot provide definitive comparative effectiveness estimates. The proposed staging framework should therefore be interpreted as an evidence-informed clinical framework intended to support individualized decision-making rather than as a validated guideline or prescriptive treatment algorithm.

## Conclusions

Current evidence supports a transition from stage-based dogma toward a more individualized, evidence-informed approach to septic revision TKA. One-stage revision can achieve infection control comparable to two-stage exchange in carefully selected patients and may offer advantages in complications, function, and treatment burden. Two-stage exchange remains essential for biologically difficult and reconstructively complex infections but carries meaningful risks of morbidity, non-reimplantation, and prolonged disability. The 1.5-stage strategy is a promising function-preserving alternative with encouraging infection-free outcomes, although its long-term mechanical durability and optimal indications remain incompletely defined. The most appropriate contemporary approach is therefore not to consider one-stage, 1.5-stage, or two-stage revision universally superior, but to match the treatment strategy to host physiology, pathogen profile, local soft-tissue and bone conditions, reconstructive feasibility, timing, and patient goals. The framework proposed in this review should be regarded as hypothesis-generating and evidence-informed rather than as a validated clinical algorithm. Future prospective, knee-specific studies should evaluate these decision domains using standardized definitions of infection control and treatment failure, consistent reporting of host characteristics and microbiological classifications, uniform patient-reported outcome measures, and longer-term follow-up of 1.5-stage constructs. Such methodological standardization will be necessary before reproducible and externally validated staging algorithms can be developed.
